# Supplementary material for: In Vitro Efficacy of Isobutyl Cyanoacrylate Nanoparticles against Fish Bacterial Pathogens and Selection Preference by Rainbow Trout (Oncorhynchus mykiss)
Source: Microorganisms. 2023 Nov 28;11(12):2877. doi: 10.3390/microorganisms11122877 (PMC10745873; doi:10.3390/microorganisms11122877)
Supplement: Supplementary file 1 [file microorganisms-11-02877-s001.zip › microorganisms-2639750-supplementary.pdf]

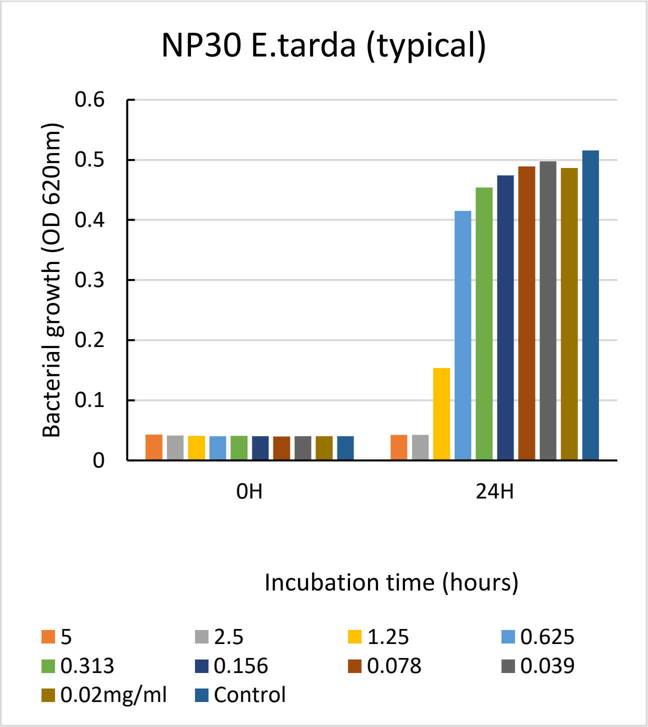
NP30 E.tarda (typical)
Bacterial growth (OD 620nm)
0.6
0.5
0.4
0.3
0.2
0.1
0
0H
24H
Incubation time (hours)
5
2.5
1.25
0.625
0.313
0.156
0.078
0.039
0.02mg/ml
Control

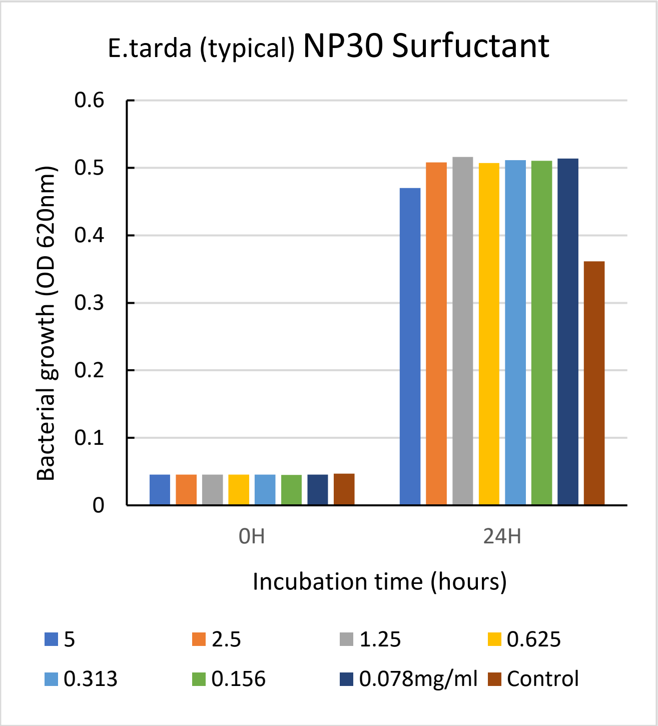
E.tarda (typical) NP30 Surfactant
Bacterial growth (OD 620nm)
0.6
0.5
0.4
0.3
0.2
0.1
0
0H
24H
Incubation time (hours)
5
2.5
1.25
0.625
0.313
0.156
0.078mg/ml
Control

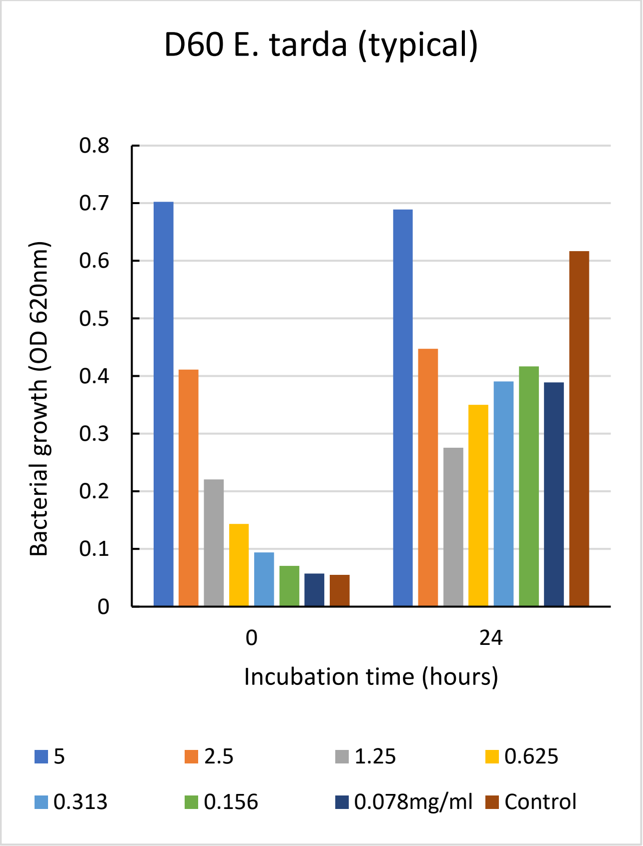
D60 E. tarda (typical)
Bacterial growth (OD 620nm)
0.8
0.7
0.6
0.5
0.4
0.3
0.2
0.1
0
0
24
Incubation time (hours)
5
2.5
1.25
0.625
0.313
0.156
0.078mg/ml
Control

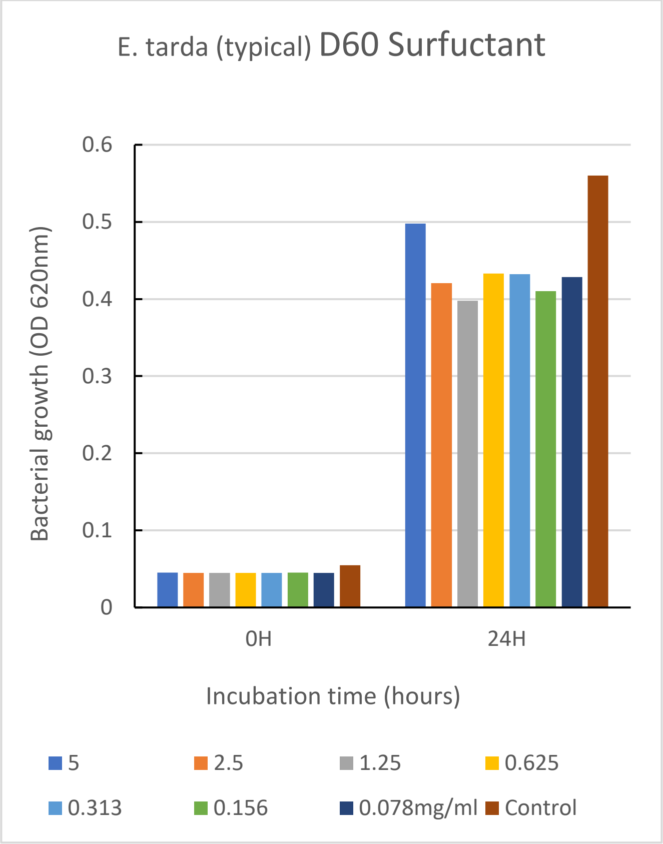
E. tarda (typical) D60 Surfactant
Bacterial growth (OD 620nm)
0.6
0.5
0.4
0.3
0.2
0.1
0
0H
24H
Incubation time (hours)
5
2.5
1.25
0.625
0.313
0.156
0.078mg/ml
Control

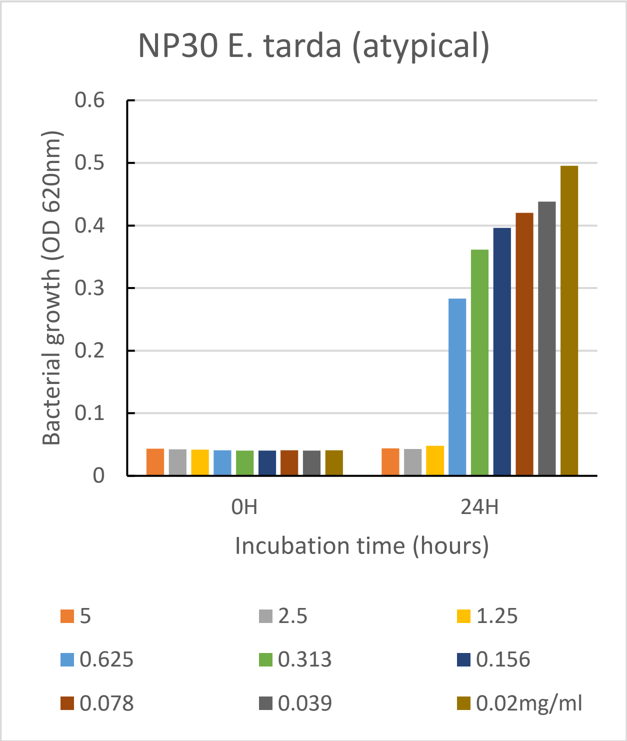
NP30 E. tarda (atypical)
Bacterial growth (OD 620nm)
0.6
0.5
0.4
0.3
0.2
0.1
0
0H
24H
Incubation time (hours)
5
2.5
1.25
0.625
0.313
0.156
0.078
0.039
0.02mg/ml

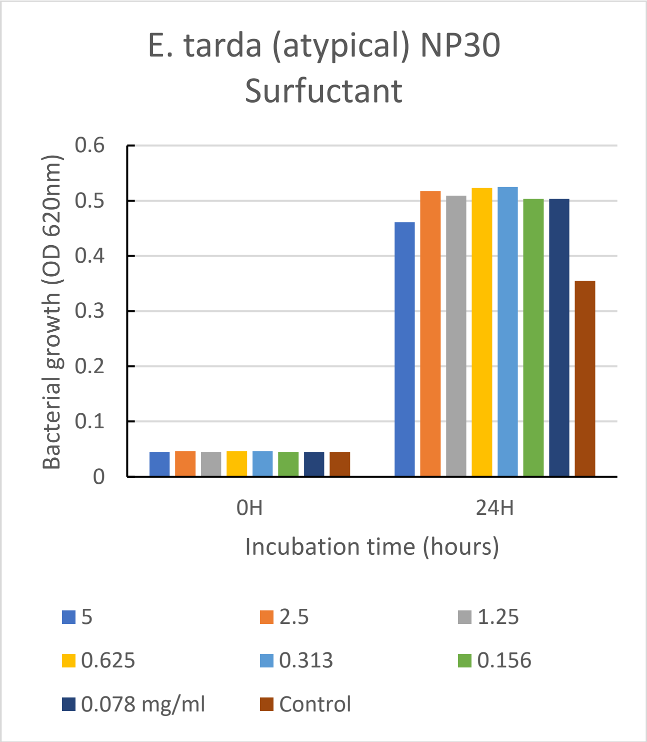
E. tarda (atypical) NP30 Surfactant
Bacterial growth (OD 620nm)
0.6
0.5
0.4
0.3
0.2
0.1
0
0H
24H
Incubation time (hours)
5
2.5
1.25
0.625
0.313
0.156
0.078 mg/ml
Control

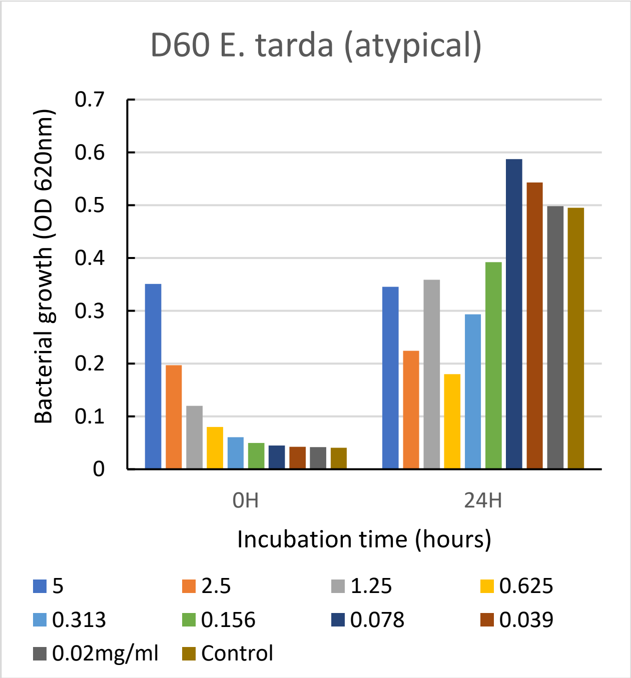
D60 E. tarda (atypical)
Bacterial growth (OD 620nm)
0.7
0.6
0.5
0.4
0.3
0.2
0.1
0
0H
24H
Incubation time (hours)
5
2.5
1.25
0.625
0.313
0.156
0.078
0.039
0.02mg/ml
Control

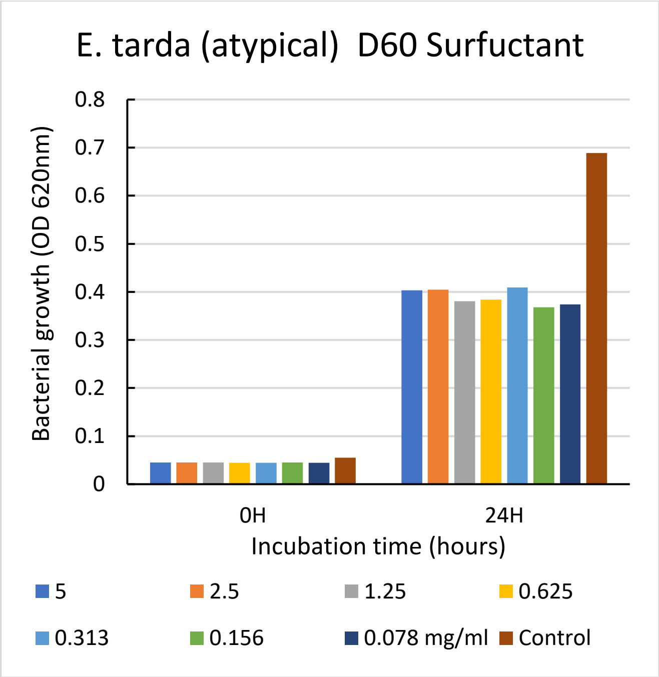
E. tarda (atypical) D60 Surfactant
Bacterial growth (OD 620nm)
0.8
0.7
0.6
0.5
0.4
0.3
0.2
0.1
0
0H
24H
Incubation time (hours)
5
2.5
1.25
0.625
0.313
0.156
0.078 mg/ml
Control

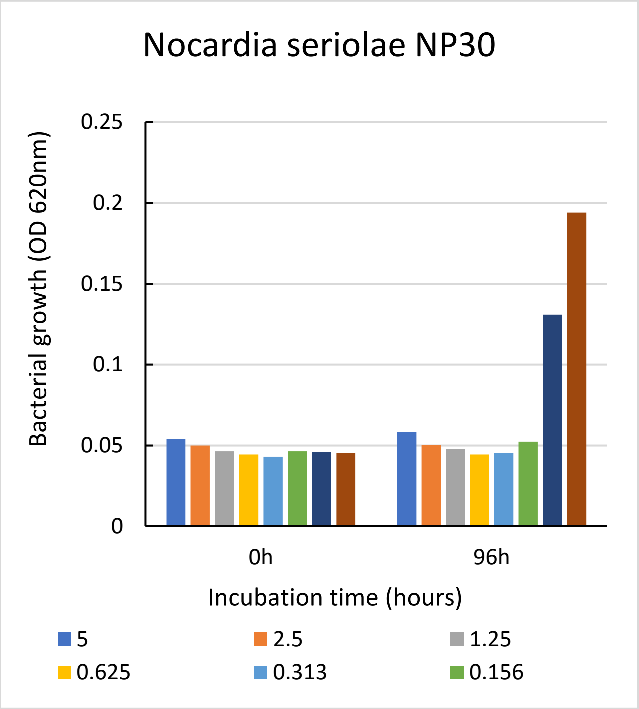
Nocardia seriolae NP30
Bacterial growth (OD 620nm)
0.25
0.2
0.15
0.1
0.05
0
0h
96h
Incubation time (hours)
5
2.5
1.25
0.625
0.313
0.156

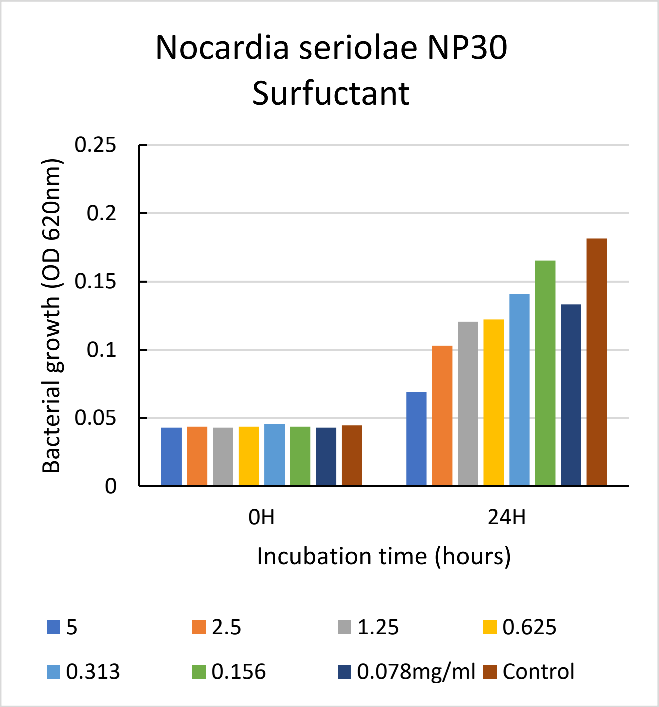
Nocardia seriolae NP30 Surfactant
Bacterial growth (OD 620nm)
0.25
0.2
0.15
0.1
0.05
0
0H
24H
Incubation time (hours)
5
2.5
1.25
0.625
0.313
0.156
0.078mg/ml
Control

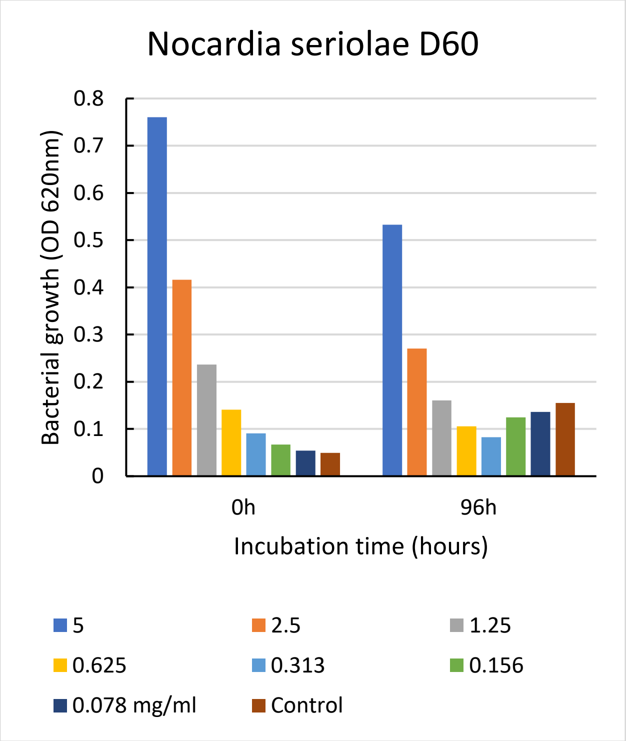
Nocardia seriolae D60
Bacterial growth (OD 620nm)
0.8
0.7
0.6
0.5
0.4
0.3
0.2
0.1
0
0h
96h
Incubation time (hours)
5
2.5
1.25
0.625
0.313
0.156
0.078 mg/ml
Control

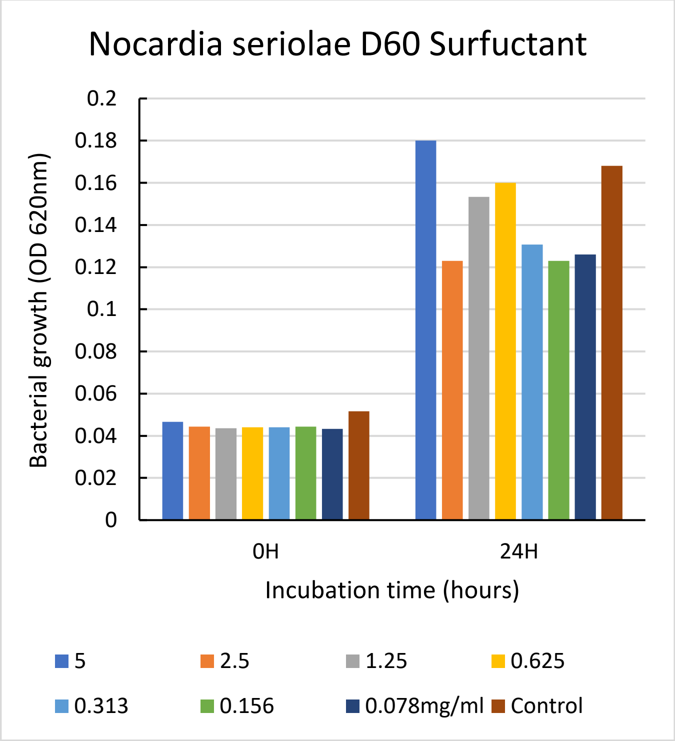
Nocardia seriolae D60 Surfactant
Bacterial growth (OD 620nm)
0.2
0.18
0.16
0.14
0.12
0.1
0.08
0.06
0.04
0.02
0
0H
24H
Incubation time (hours)
5
2.5
1.25
0.625
0.313
0.156
0.078mg/ml
Control

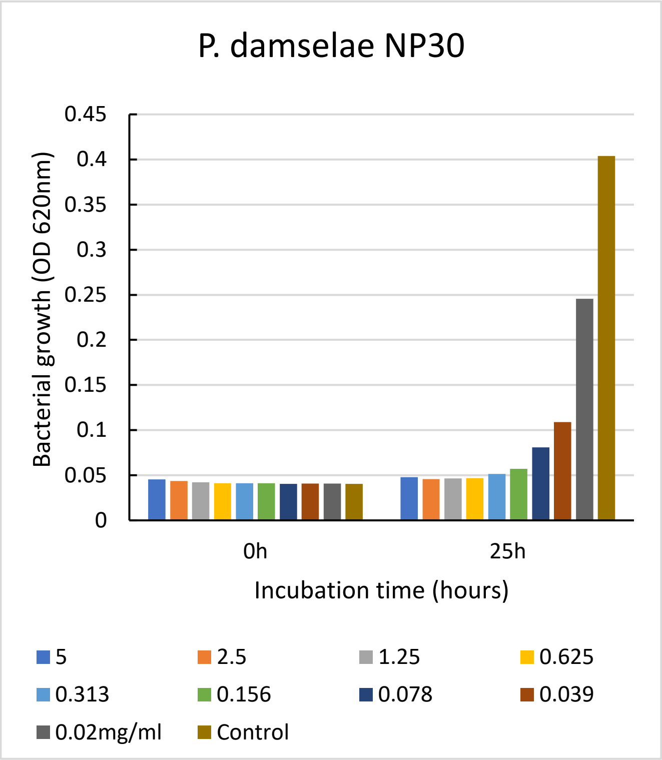
P. damselae NP30
Bacterial growth (OD 620nm)
0.45
0.4
0.35
0.3
0.25
0.2
0.15
0.1
0.05
0
0h
25h
Incubation time (hours)
5
2.5
1.25
0.625
0.313
0.156
0.078
0.039
0.02mg/ml
Control

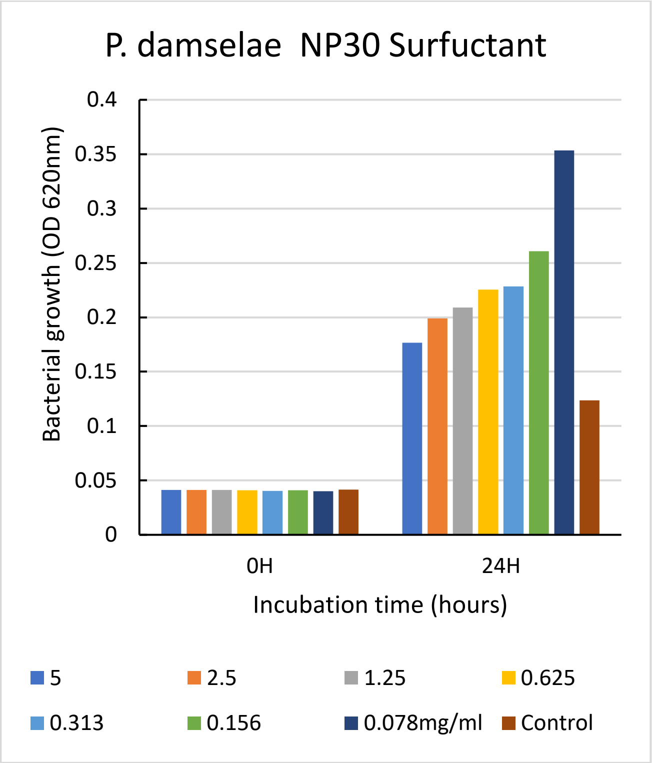
P. damselae NP30 Surfuctant
Bacterial growth (OD 620nm)
0.4
0.35
0.3
0.25
0.2
0.15
0.1
0.05
0
0H
24H
Incubation time (hours)
5
2.5
1.25
0.625
0.313
0.156
0.078mg/ml
Control

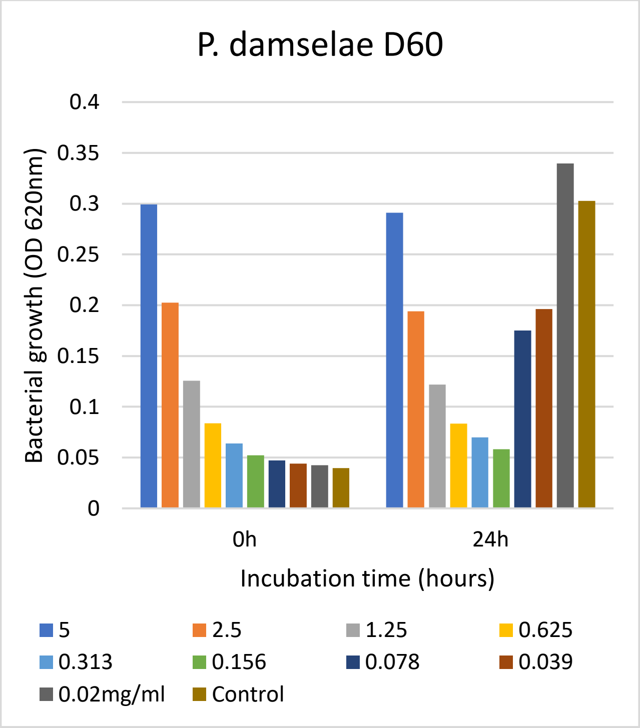
P. damselae D60
Bacterial growth (OD 620nm)
0.4
0.35
0.3
0.25
0.2
0.15
0.1
0.05
0
0h
24h
Incubation time (hours)
5
2.5
1.25
0.625
0.313
0.156
0.078
0.039
0.02mg/ml
Control

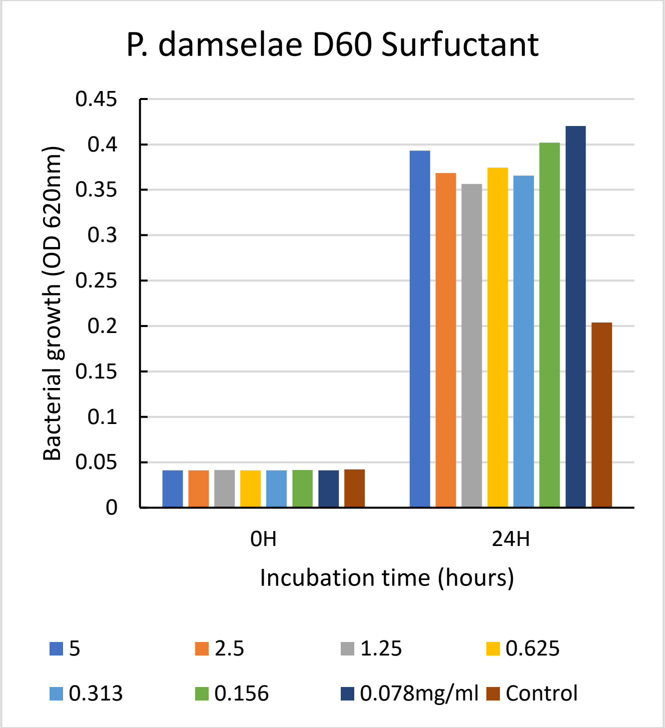
P. damselae D60 Surfuctant
Bacterial growth (OD 620nm)
0.45
0.4
0.35
0.3
0.25
0.2
0.15
0.1
0.05
0
0H
24H
Incubation time (hours)
5
2.5
1.25
0.625
0.313
0.156
0.078mg/ml
Control

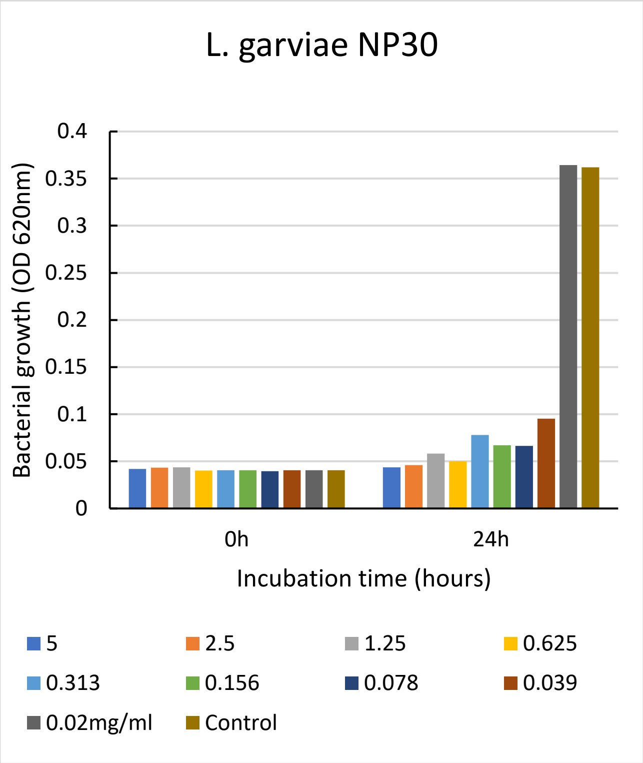
L. garviae NP30
Bacterial growth (OD 620nm)
0.4
0.35
0.3
0.25
0.2
0.15
0.1
0.05
0
0h
24h
Incubation time (hours)
5
2.5
1.25
0.625
0.313
0.156
0.078
0.039
0.02mg/ml
Control

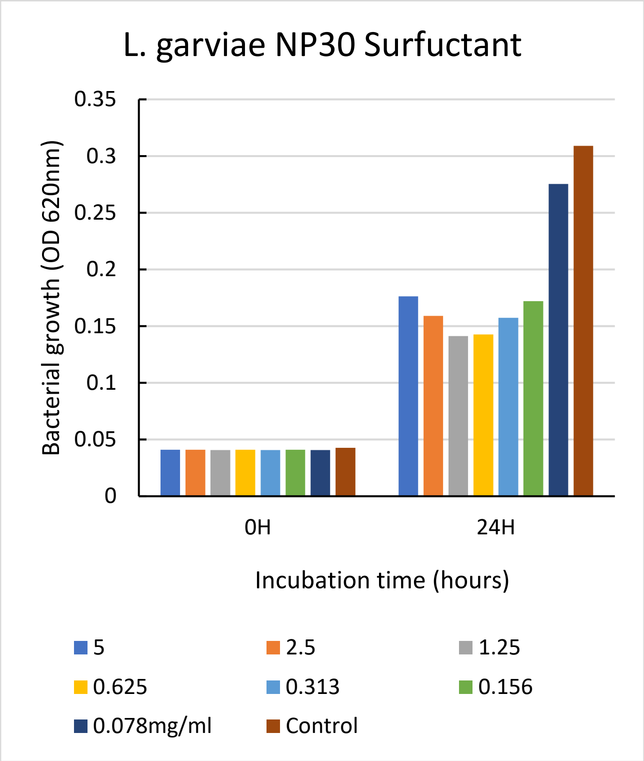
L. garviae NP30 Surfuctant
Bacterial growth (OD 620nm)
0.35
0.3
0.25
0.2
0.15
0.1
0.05
0
0H
24H
Incubation time (hours)
5
2.5
1.25
0.625
0.313
0.156
0.078mg/ml
Control

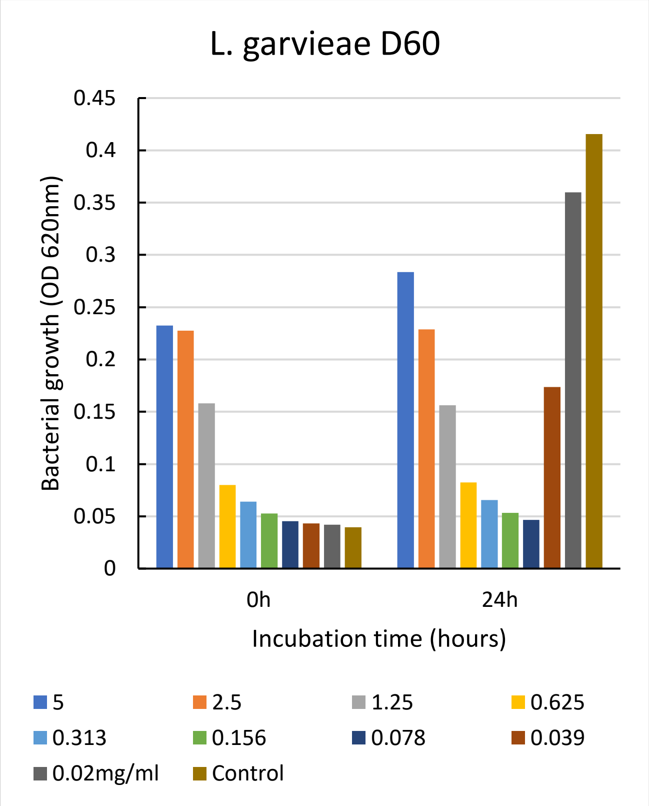
L. garvieae D60
Bacterial growth (OD 620nm)
0.45
0.4
0.35
0.3
0.25
0.2
0.15
0.1
0.05
0
0h
24h
Incubation time (hours)
5
2.5
1.25
0.625
0.313
0.156
0.078
0.039
0.02mg/ml
Control

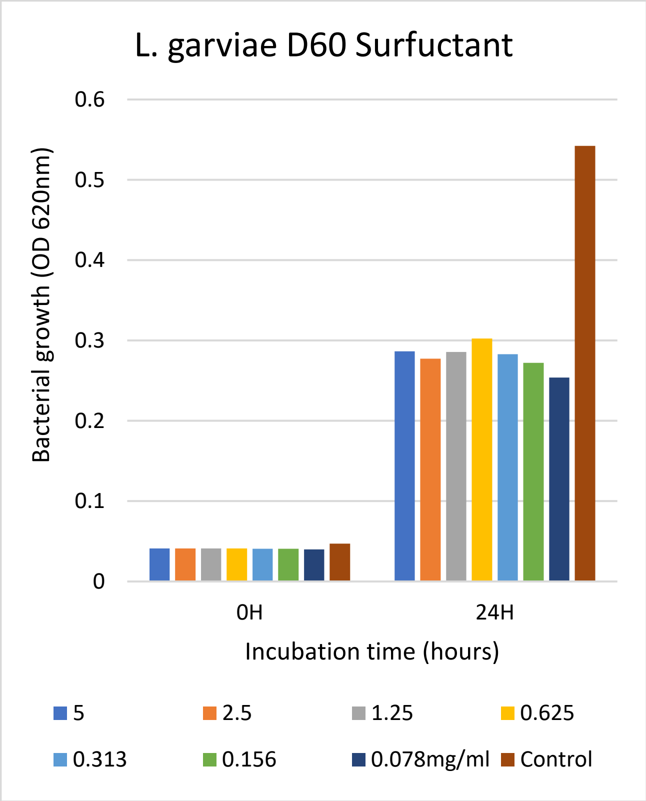
L. garviae D60 Surfuctant
Bacterial growth (OD 620nm)
0.6
0.5
0.4
0.3
0.2
0.1
0
0H
24H
Incubation time (hours)
5
2.5
1.25
0.625
0.313
0.156
0.078mg/ml
Control

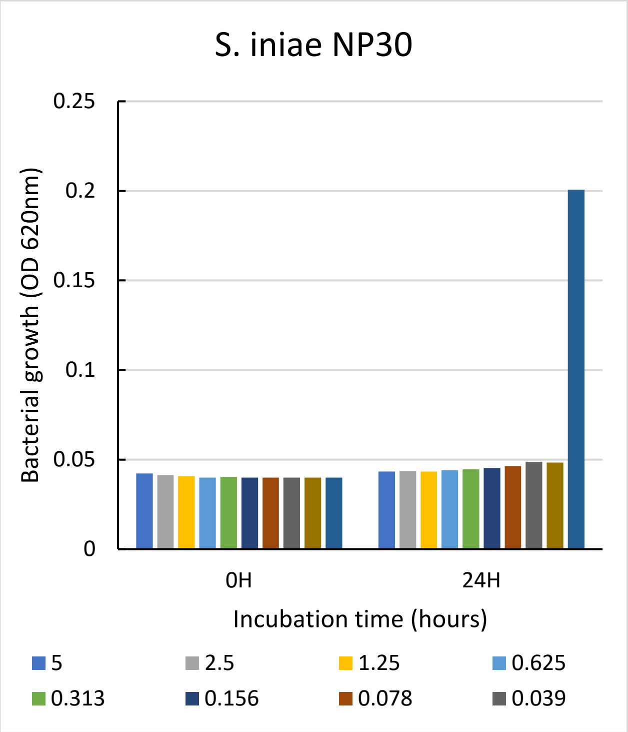
S. iniae NP30
Bacterial growth (OD 620nm)
0.25
0.2
0.15
0.1
0.05
0
0H
24H
Incubation time (hours)
5
2.5
1.25
0.625
0.313
0.156
0.078
0.039

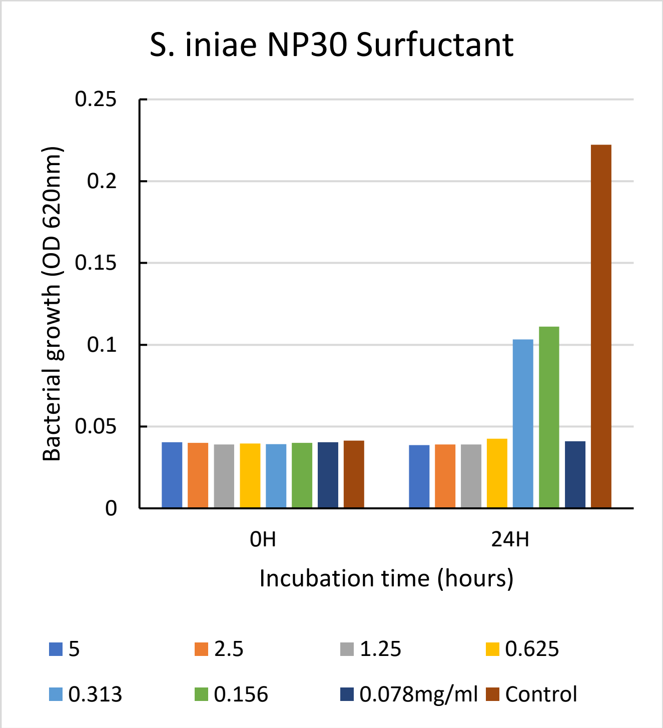
S. iniae NP30 Surfactant
Bacterial growth (OD 620nm)
0.25
0.2
0.15
0.1
0.05
0
0H
24H
Incubation time (hours)
5
2.5
1.25
0.625
0.313
0.156
0.078mg/ml
Control

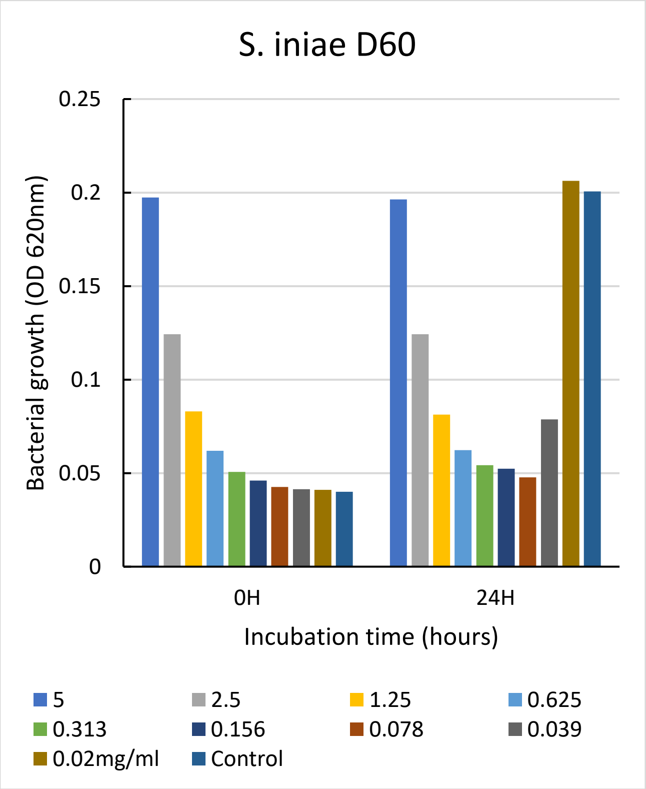
S. iniae D60
Bacterial growth (OD 620nm)
0.25
0.2
0.15
0.1
0.05
0
0H
24H
Incubation time (hours)
5
2.5
1.25
0.625
0.313
0.156
0.078
0.039
0.02mg/ml
Control

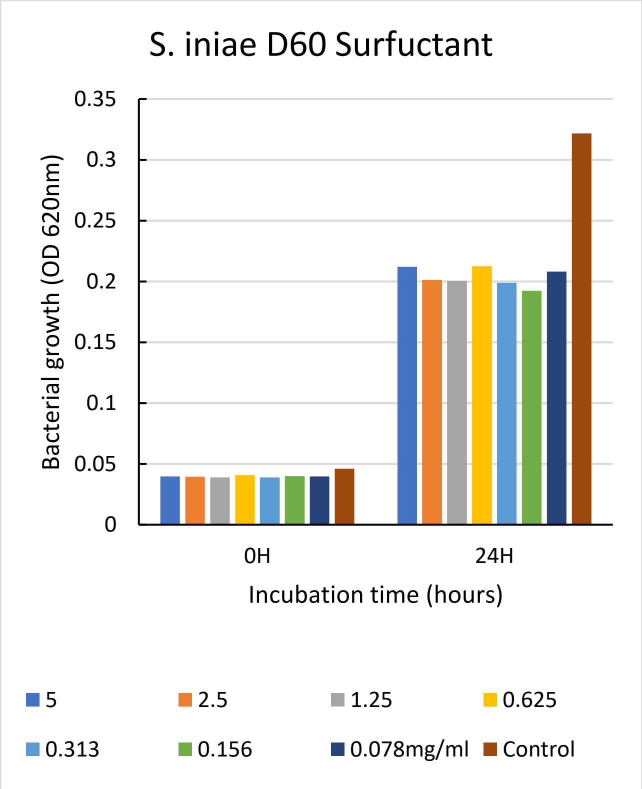
S. iniae D60 Surfactant
Bacterial growth (OD 620nm)
0.35
0.3
0.25
0.2
0.15
0.1
0.05
0
0H
24H
Incubation time (hours)
5
2.5
1.25
0.625
0.313
0.156
0.078mg/ml
Control

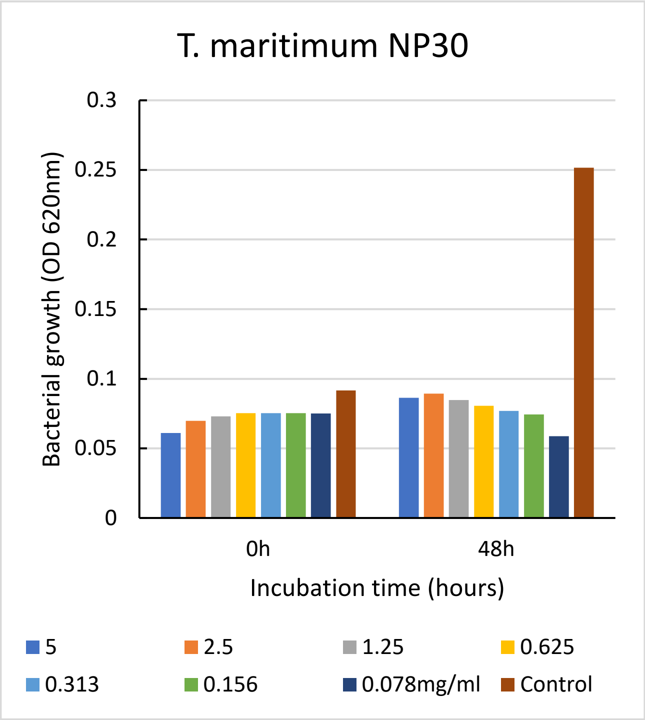
T. maritimum NP30
Bacterial growth (OD 620nm)
0.3
0.25
0.2
0.15
0.1
0.05
0
0h
48h
Incubation time (hours)
5
2.5
1.25
0.625
0.313
0.156
0.078mg/ml
Control

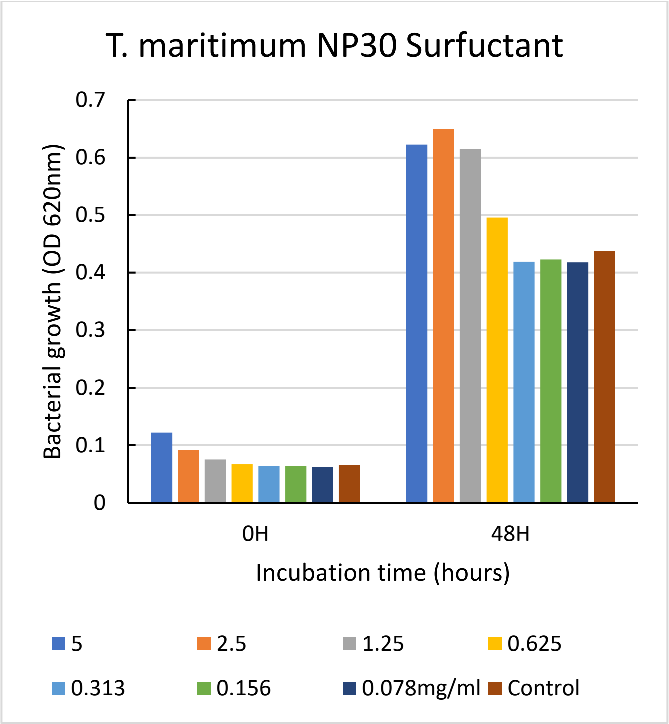
T. maritimum NP30 Surfuctant
Bacterial growth (OD 620nm)
0.7
0.6
0.5
0.4
0.3
0.2
0.1
0
0H
48H
Incubation time (hours)
5
2.5
1.25
0.625
0.313
0.156
0.078mg/ml
Control

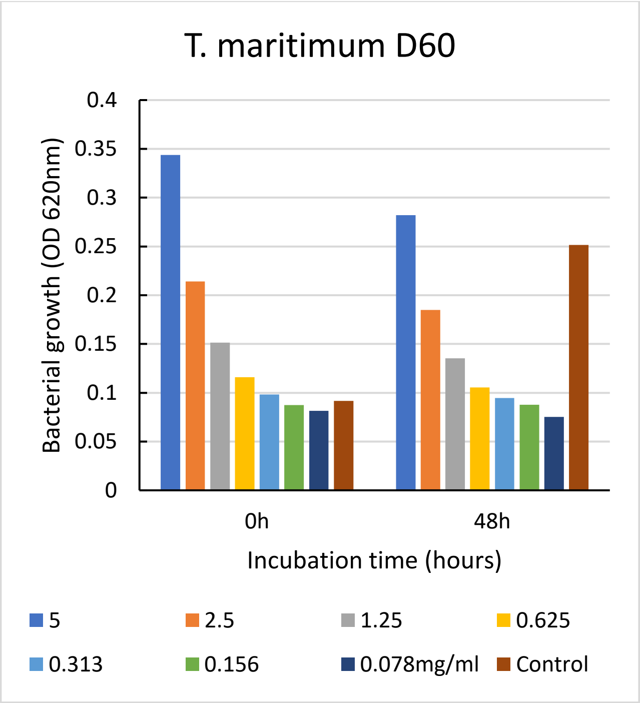
T. maritimum D60
Bacterial growth (OD 620nm)
0.4
0.35
0.3
0.25
0.2
0.15
0.1
0.05
0
0h
48h
Incubation time (hours)
5
2.5
1.25
0.625
0.313
0.156
0.078mg/ml
Control

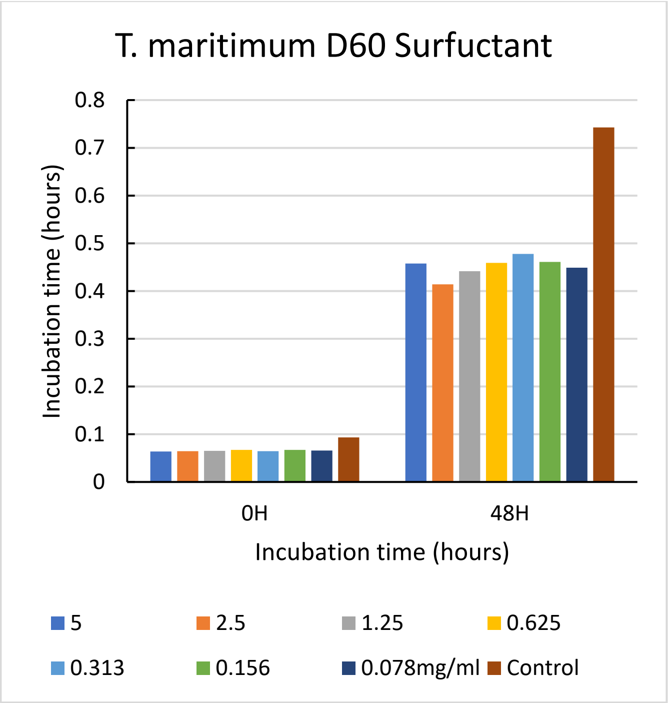
T. maritimum D60 Surfuctant
Incubation time (hours)
0.8
0.7
0.6
0.5
0.4
0.3
0.2
0.1
0
0H
48H
Incubation time (hours)
5
2.5
1.25
0.625
0.313
0.156
0.078mg/ml
Control

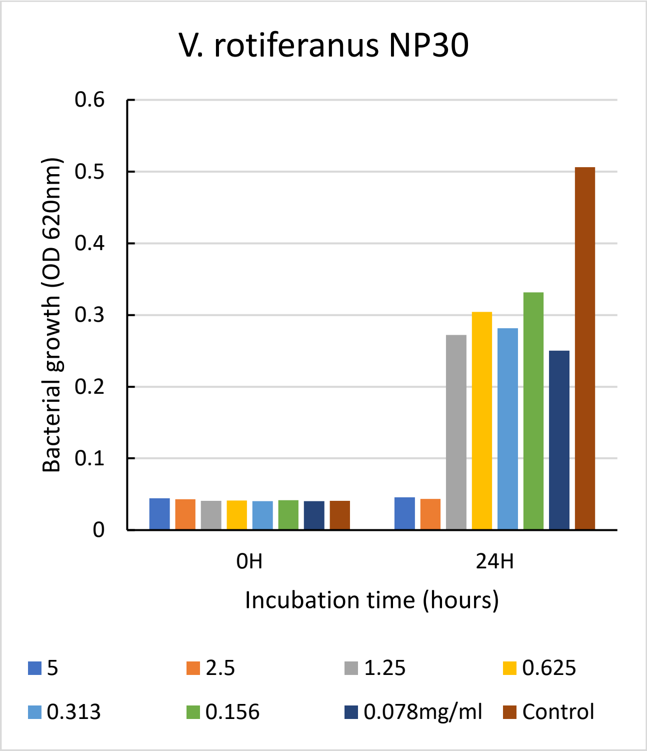
V. rotiferanus NP30
Bacterial growth (OD 620nm)
0.6
0.5
0.4
0.3
0.2
0.1
0
0H
24H
Incubation time (hours)
5
2.5
1.25
0.625
0.313
0.156
0.078mg/ml
Control

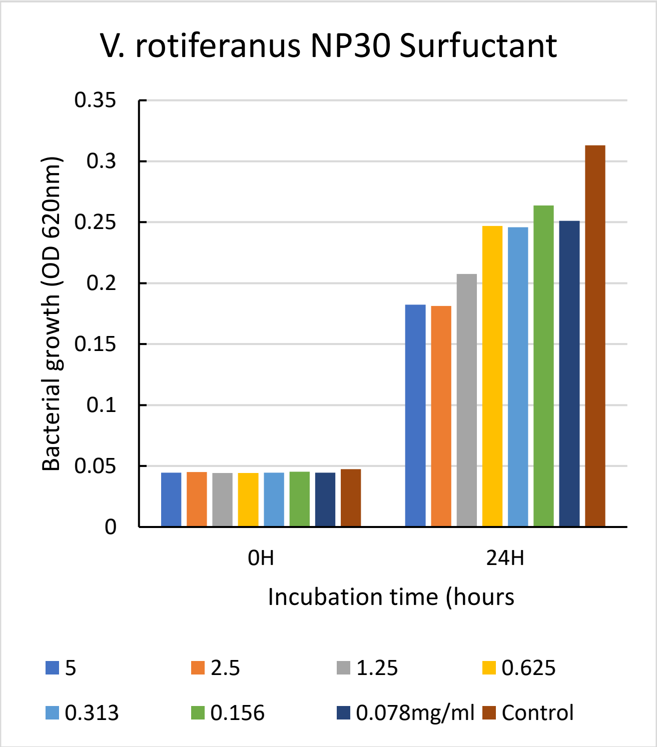
V. rotiferanus NP30 Surfuctant
Bacterial growth (OD 620nm)
0.35
0.3
0.25
0.2
0.15
0.1
0.05
0
0H
24H
Incubation time (hours
5
2.5
1.25
0.625
0.313
0.156
0.078mg/ml
Control

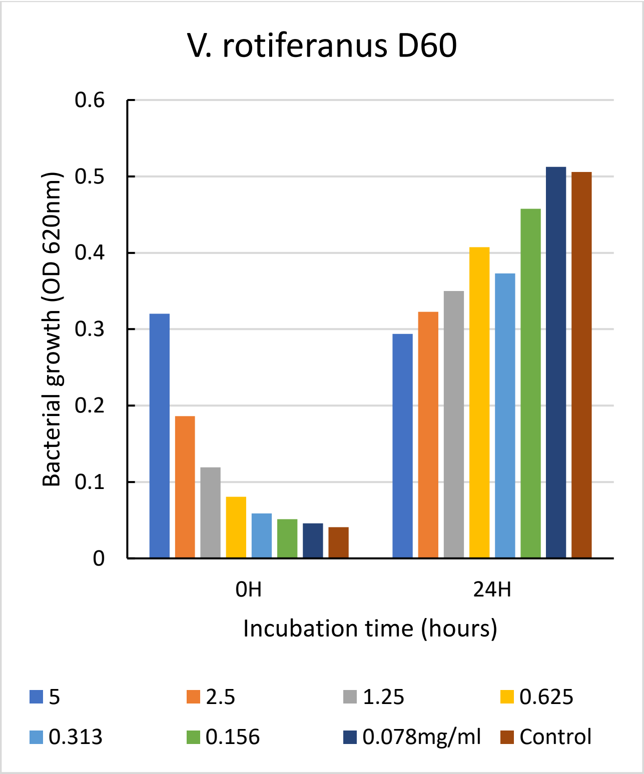
V. rotiferanus D60
Bacterial growth (OD 620nm)
0.6
0.5
0.4
0.3
0.2
0.1
0
0H
24H
Incubation time (hours)
5
2.5
1.25
0.625
0.313
0.156
0.078mg/ml
Control

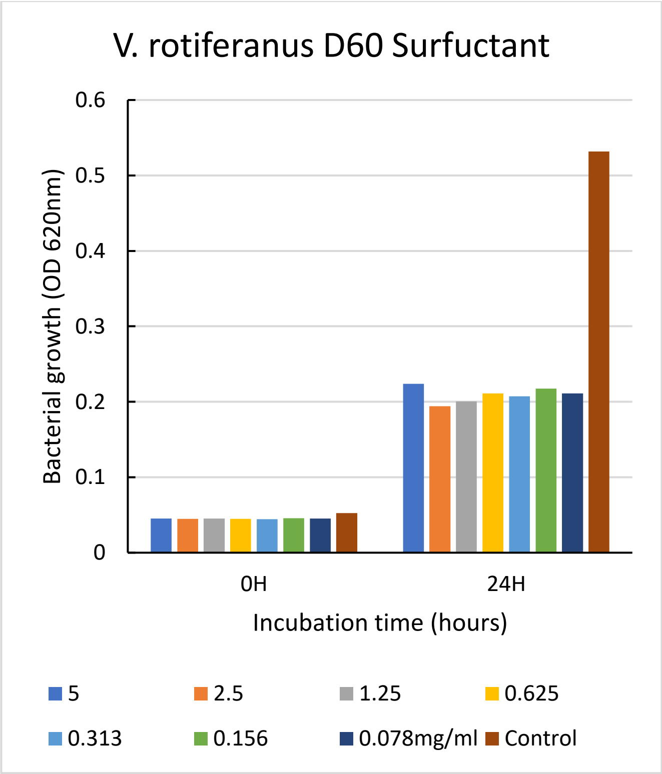
V. rotiferanus D60 Surfuctant
Bacterial growth (OD 620nm)
0.6
0.5
0.4
0.3
0.2
0.1
0
0H
24H
Incubation time (hours)
5
2.5
1.25
0.625
0.313
0.156
0.078mg/ml
Control

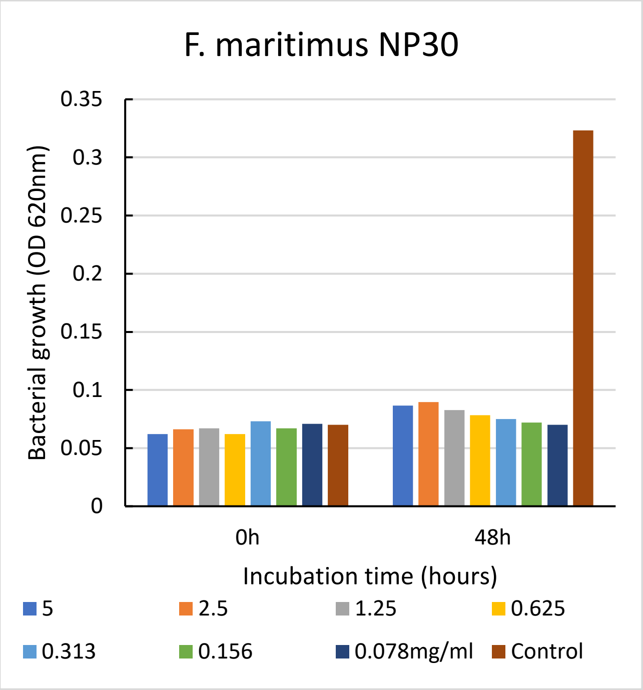
F. maritimus NP30
Bacterial growth (OD 620nm)
0.35
0.3
0.25
0.2
0.15
0.1
0.05
0
0h
48h
Incubation time (hours)
5
2.5
1.25
0.625
0.313
0.156
0.078mg/ml
Control

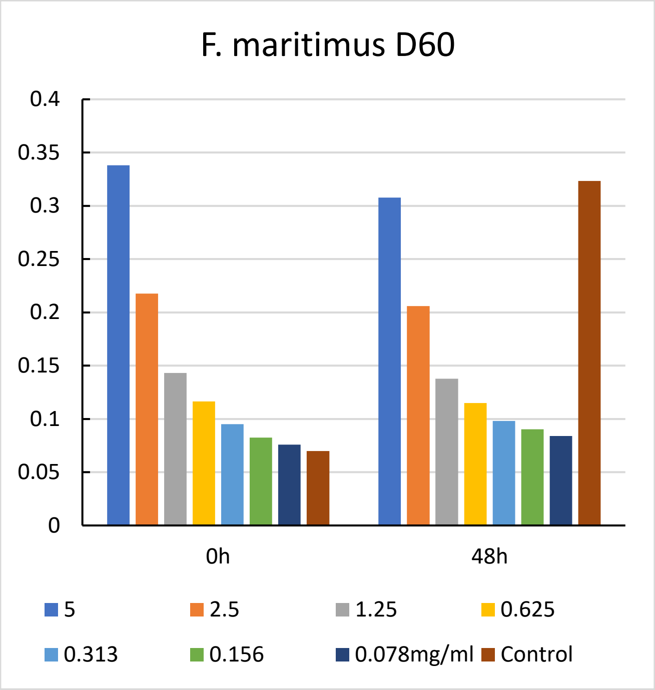
F. maritimus D60
0.4
0.35
0.3
0.25
0.2
0.15
0.1
0.05
0
0h
48h
5
2.5
1.25
0.625
0.313
0.156
0.078mg/ml
Control

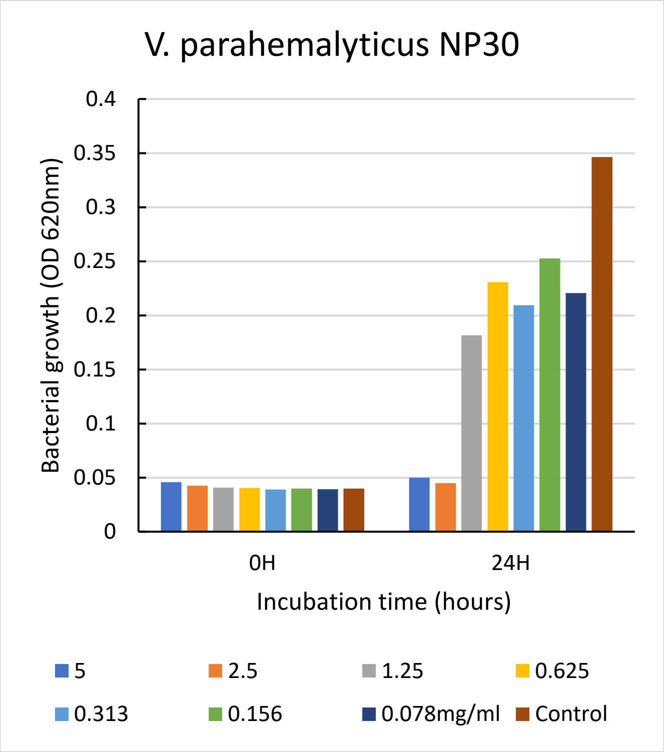
V. parahemalyticus NP30
Bacterial growth (OD 620nm)
0.4
0.35
0.3
0.25
0.2
0.15
0.1
0.05
0
0H
24H
Incubation time (hours)
5
2.5
1.25
0.625
0.313
0.156
0.078mg/ml
Control

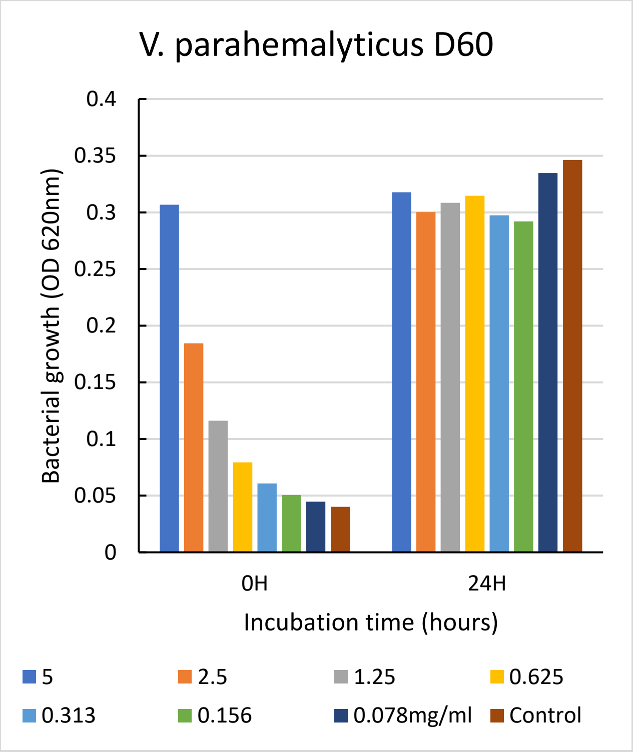
V. parahemalyticus D60
Bacterial growth (OD 620nm)
0.4
0.35
0.3
0.25
0.2
0.15
0.1
0.05
0
0H
24H
Incubation time (hours)
5
2.5
1.25
0.625
0.313
0.156
0.078mg/ml
Control

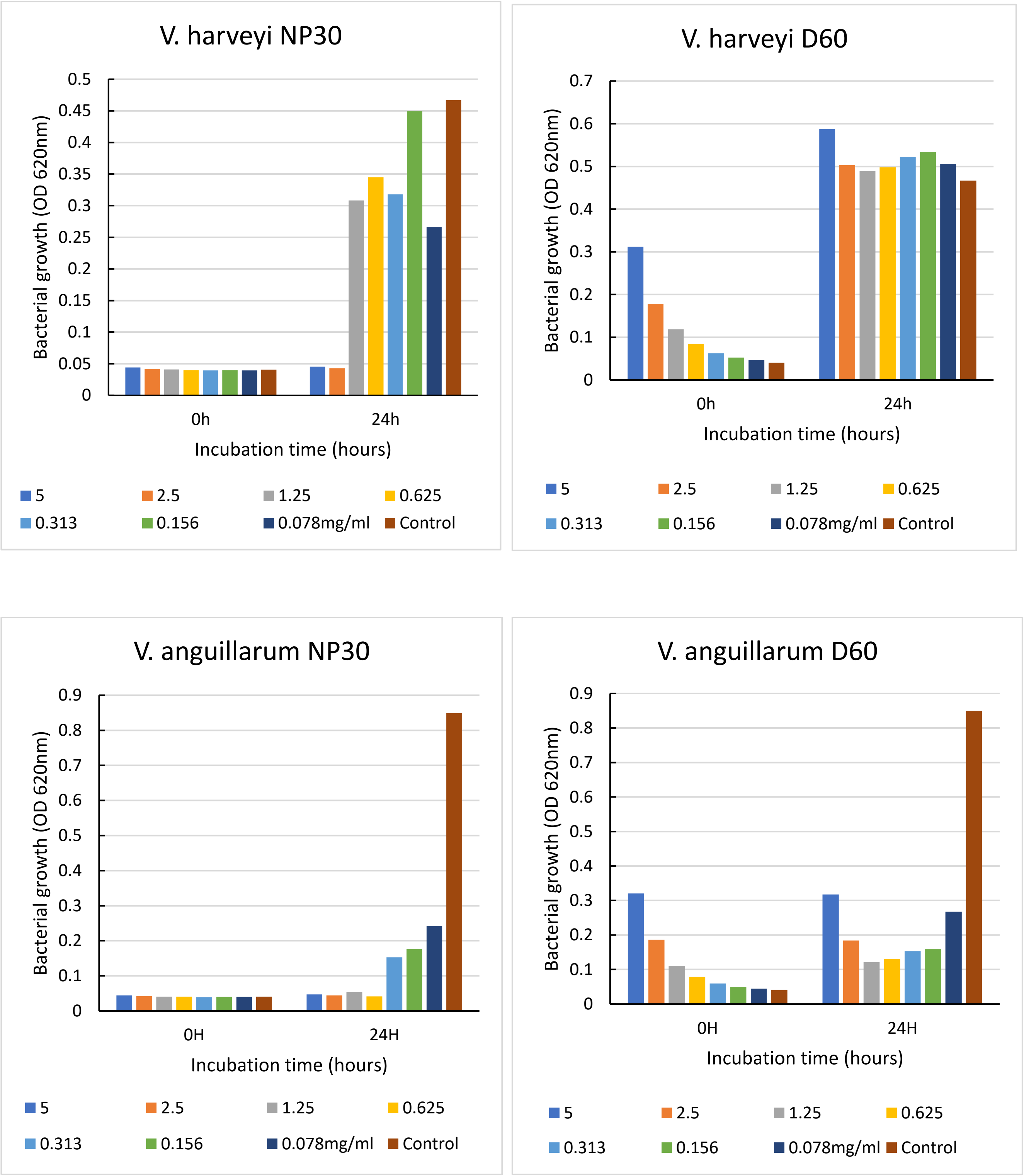

Figure S1: Bacteria growth before (0H) and after incubation (24H/48H or 96H) of MIC assays for each bacteria strain using the isobutyl-cyanoacrylate nanoparticles (D60 & NP30) and their respective dispersants.

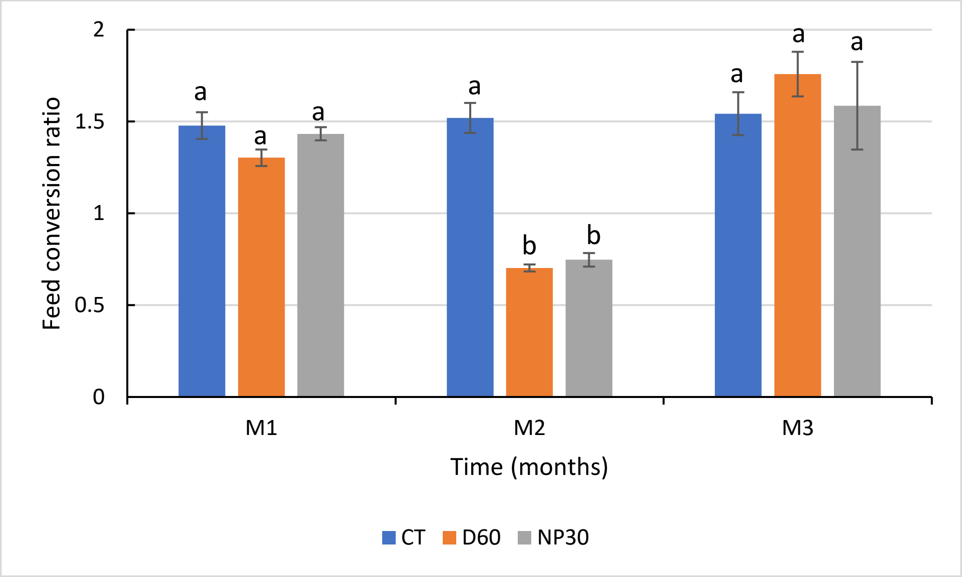

Figure S2: Monthly feed conversion ratio per treatment where different letter superscripts, *a*, and *b*, indicate significant differences between treatments and or sampling time ($P < 0.05$). CT = Control group; M1, M2 & M3 = Months 1,2 & 3. All data were expressed as means ± SEM, n = 2.
